# Importance of modifiable non-radiographic functional parameters for adult spinal deformity

**DOI:** 10.1038/s41598-024-54854-8

**Published:** 2024-03-22

**Authors:** Kozaburo Mizutani, Tetsuya Kobayashi, Issei Senoo, Mutsuya Shimizu, Hiroki Okayasu

**Affiliations:** Department of Orthopaedic Surgery, Asahikawa Medical University, 2-1E Midorigaoka, Asahikawa, Hokkaido 0788510 Japan

**Keywords:** Physiology, Anatomy, Medical research

## Abstract

We clarified non-radiographic physical parameters associated with the severity of adult spinal deformity (ASD) using community-dwelling adult volunteers. They were subjected to upright entire spine radiographs for standard radiographic parameters and the number of sagittal modifiers of SRS-Schwab ASD classification (Schwab-SM). Clinical evaluations included isometric muscle strength of trunk extensor (TEX), trunk flexor (TFL), quadriceps femoris (QF), gluteus maximus, and iliopsoas; range of motion (ROM) of hip, knee, ankle, and active back extension (BET); SF36 physical component score (PCS), VAS for back and knee pain, and the degree of ambulatory kyphosis (dTIA). Each muscle strength was calibrated by body weight (BW) and expressed as BW ratio. According to our previous study, dTIA ≥ 7.6° was defined as pathological and dTIA ≤ 3.5° as normal. A final total of 409 female volunteers were included, and their demographics were; age 67.0 ± 5.5 years, Schwab-SM 2.1 ± 1.8, TEX 0.90 ± 0.33BW, TFL 0.48 ± 0.15BW, QF 0.45 ± 0.19BW, PCS 33.5 ± 6.5. Subjects were classified as clinical ASD group (cASD, n = 10) with PCS ≤ 27(mean-1SD) and pathological dTIA, robust group (n = 19) with PCS ≥ 40 (mean + 1SD) and normal dTIA, and the rest (non-cASD, n = 338). Statistical analyses showed significant differences in TEX, TFL, QF, knee extension (KEX), and BET between robust and cASD, and the mean values of robust group (TEX ≥ 1.1BW, TFL ≥ 0.5BW, QF ≥ 0.5BW, KEX ≥ 0° and BET ≥ 14 cm) were used as ‘ASD-MJ’ index. Subjects with fully achieving ASD-MJ goals showed significantly better radiographic and clinical outcomes than those with unmet goals. In conclusion, upon prescribing conservative or physical therapies for ASD patients, modifiable clinical goals should be clarified, and ASD-MJ could be a benchmark.

## Introduction

Radiographic evaluation of adult spinal deformity (ASD) has been developing over the past decades, emphasizing the importance of pelvic parameters and sagittal balance, prompted the understanding of normal spinopelvic alignment; however, radiographic normative values are only one aspect of multifaceted degenerative conditions^5,10^. Non-radiographic characteristics such as the extent of muscle weakness or joint contractures in spinopelvic or lower extremity joints are yet to be answered. Recent prospective studies indicated better outcomes with surgical treatment over conservative treatment for ASD^1–3^, however, detailed protocols for conservative treatment, such as targeted goals of physical therapy, could not be demonstrated. The purpose of this study was to clarify non-radiographic factors associated with the severity of sagittal balance in ASD.

## Methods

This study was a part of our ongoing longitudinal cohort study, Asahikawa observational study of Spinal Aging in Prospective cohort (the ASAP study), which has been recruiting adult volunteers from population register since 1983. Follow-up study has been conducted since 1997 according to following criteria; included if females over 40 years of age, healthy enough to walk independently to attend our program, available baseline and follow-up whole spine radiographs, and submitted written informed consent; excluded if history of spinal arthrodesis or joint replacement surgery, severe systemic or orthopaedic pathology requiring hospitalization, or repetitive medical consultation.

All methods were performed in accordance with the relevant guidelines and regulations.

**Figure 1:**
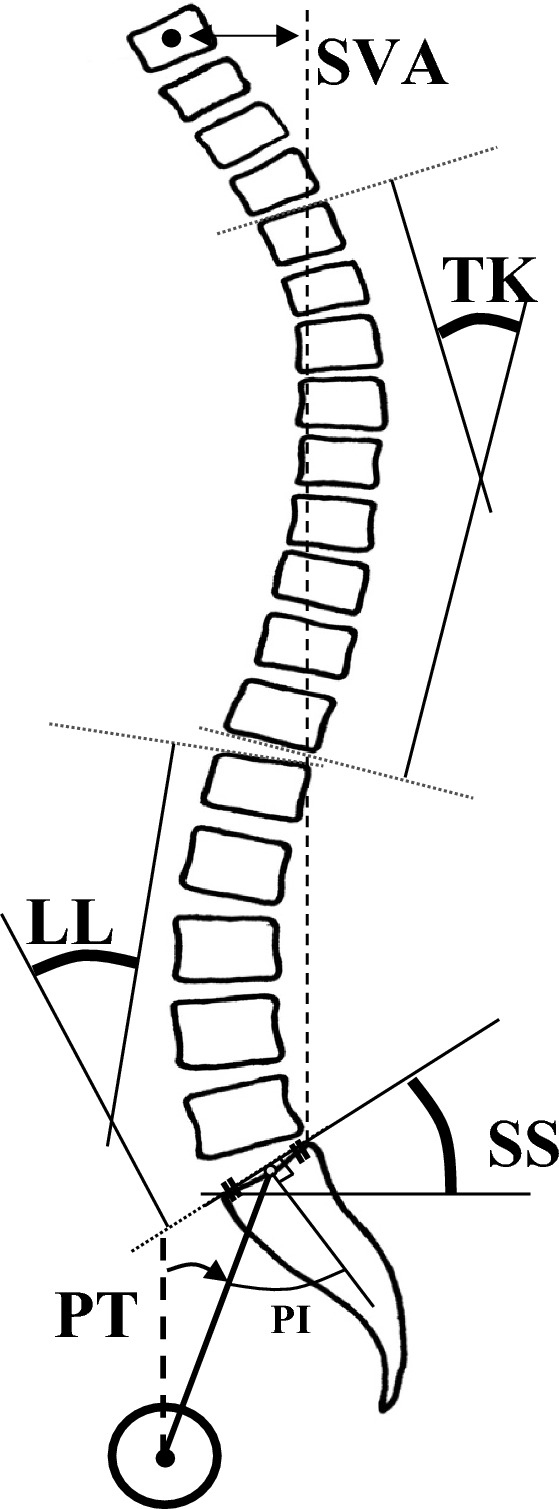
Radiographic measurements thoracic kyphosis (TK, between upper endplate of T4 and lower endplate of T12), lumbar lordosis (LL, between upper endplate of L1 and S1), sagittal vertical axis (SVA, distance of plumb lines through C7 and S1 posterior edge), sacral slope (SS, between upper sacral endplate and horizontal reference), pelvic tilt (PT, between the line through center of femoral head and midpoint of sacral table and vertical reference), pelvic incidence (PI, between the line through center of femoral head and midpoint of sacral table and the line perpendicular to sacral table), and percent slip (length of vertebral displacement divided by the length of vertebral endplate below; S/V in percent). TK thoracic kyphosis, LL lumbar lordosis, SVA sagittal vertical axis, SS sacral slope, PT pelvic tilt, PI pelvic incidence.

Upright entire spine radiographs by the digital image software were taken for standard radiographic measurements (Fig. 1); thoracic kyphosis (TK, between upper endplate of T4 and lower endplate of T12), lumbar lordosis (LL, between upper endplate of L1 and S1), pelvic tilt (PT, between the line through center of femoral head and midpoint of sacral table, and vertical reference), pelvic incidence (PI, between the line through center of femoral head and midpoint of sacral table, and the line perpendicular to sacral table), sagittal vertical axis (SVA, distance of plumb lines through C7 and S1 posterior edge), and the number of sagittal modifiers (PI-LL < 10° = 0, 10–20° = 1, > 20° = 2; SVA < 4 cm = 0, 4–9.5 cm = 1, > 9.5 cm = 2; PT < 20° = 0, 20–30° = 1, > 30° = 2) according to Scoliosis Research Society and Schwab ASD classification (Schwab-SM)^4^.

Clinical scores were conducted by spine physicians and physical therapists, and included visual analog scale for back and knee pain, and short form 36 (SF36) physical component scale summary (PCS). SF-36 is used to assess the health-related QoL (HRQoL), and a widely used generic instrument that measures eight types of health constructs. SF-36 PCS is composed of four primary items, including the summaries of physical functioning (10 items), role limitation due to physical problems (4 items), pain (2 items), and general health (5 items). The scores range from 0 to 100, with a higher score indicating better HRQoL.

**Figure 2:**
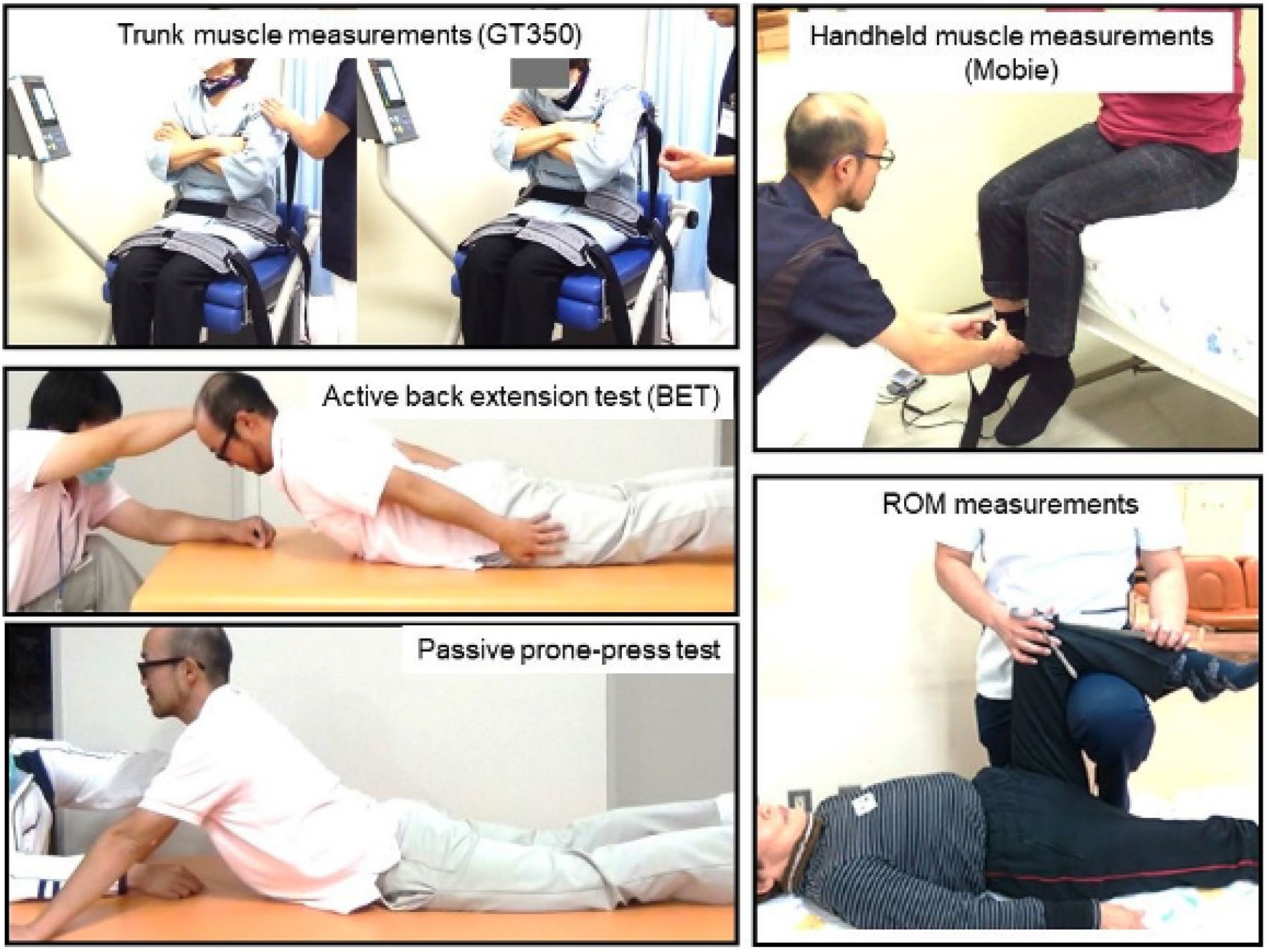
Non-radiographic measurements Trunk flexor and extensor muscle strength measured using isometric device (GT350, OG Giken Co., Japan). Lower extremities muscle strength using isometric device (a handheld dynamometer Mobie of Sakai Med Co.). Active back extension test (BET: distance of sternal notch and floor at active lumbar extension). Passive prone-press test (distance of chin and floor at maximal lumbar extension by push-up force). ROM measurements of lower extremity.

Non-radiographic measurements by physical therapists included isometric muscle strength of trunk extensor (TEX), trunk flexor (TFL), quadriceps femoris (QF), gluteus maximus, and iliopsoas, using a chair-type GT350 of OG Giken Co., or a handheld dynamometer Mobie of Sakai Med Co., and each muscle strength was calibrated with body weight (BW) and expressed as BW ratio. Joint range of motion (ROM) was also measured; hip extension/internal rotation/external rotation, knee flexion/extension (KEX), ankle dorsiflexion. Lumbar ROM was evaluated by active extension distance from the floor as back extension test (BET, distance of sternal notch and floor at maximum active lumbar extension with thighs attached to the floor) and passive extension in prone-press test (distance of chin and floor at maximum lumbar extension at push-up with thighs attached to the floor). Each participant repeated the measurement at least three times, and best scores were used as flexor and extensor strength (Fig. 2).

**Figure 3:**
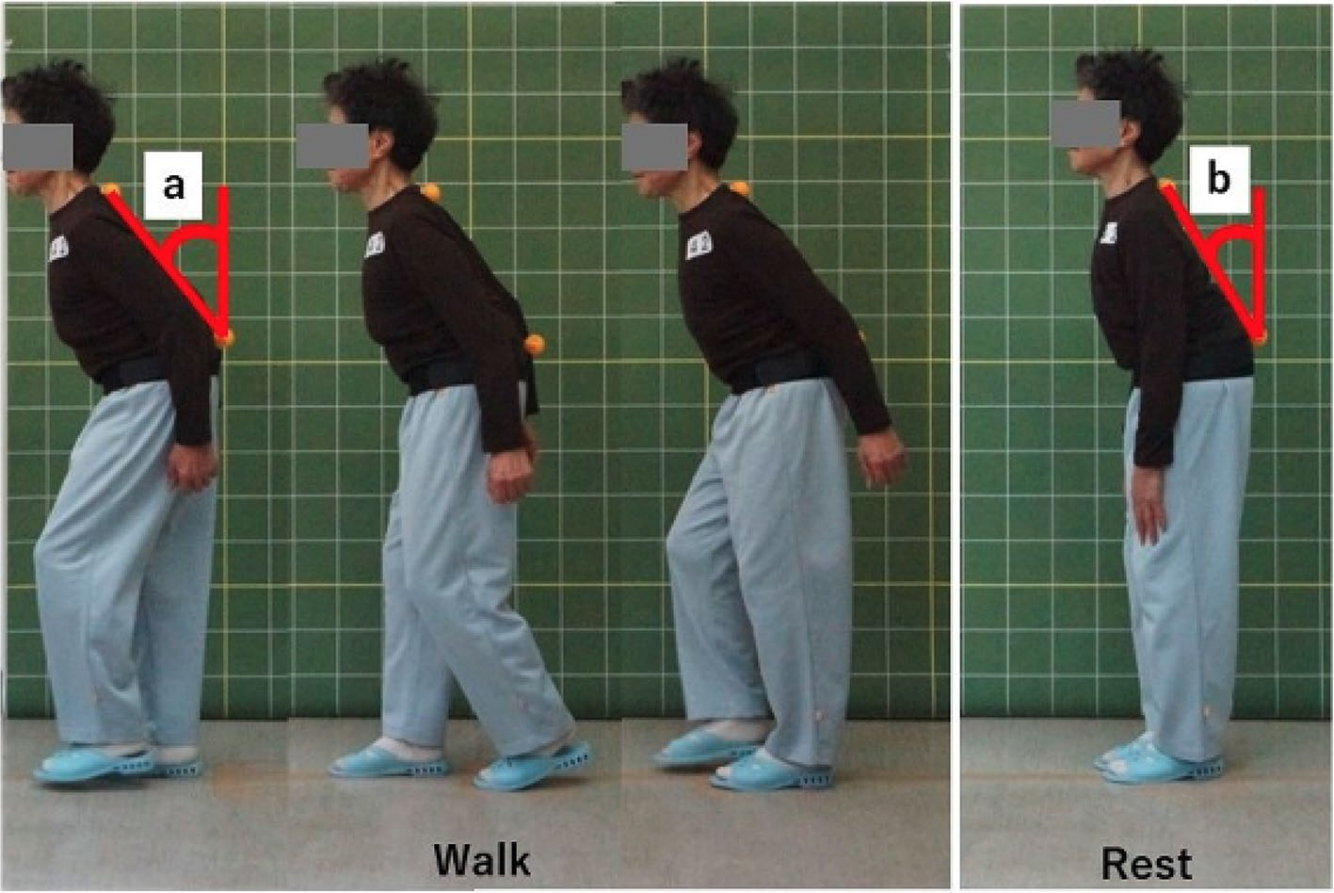
Evaluation of Ambulatory Kyphosis. Trunk inclination angle was the angle subtended by the line through surface markers attached on C7 (or on prominent cervical spinous process) and on L4 (or on intercrestal line), and the line through vertical reference. Difference of trunk inclination angle (dTIA) between 6-m walk and rest was used and defined as positive for forward inclination during walk. Ambulatory kyphosis: angle a-b (dTIA).

Since vital characteristics of ASD has been specified as dynamic deformity^5,6^, which is the worsening of deformity during walk or prolonged daily activities, we evaluated ambulatory kyphosis with surface markers and video recorders. Each participant was instructed to walk 6-m walkway with surface markers attached on C7 (or on prominent cervical spinous process) and on L4 (or on intercrestal line). Trunk inclination angle (TIA, defined as angles subtended by the line through surface markers and the vertical reference) was measured at rest and during walk, and the difference in TIA (dTIA) was recorded as ambulatory kyphosis. Details of our measurements has been previously reported^7–9^. Each participant repeated dTIA measurement at least three times, and maximum difference was used. Most subjects showed increase in trunk inclination angle at walk, and dTIA was defined as positive for forward inclination (Fig. 3).

Statistical analysis was performed using the StatView software (Abacus Concepts, Inc, Berkley, CA). Interclass comparison was done by the analysis of variance, and p value of less than 0.05 was considered as a significant difference. Institutional review board approved the study, and written informed consent was mandatory for each participant upon enrollment.

## Results

A final total of 409 female volunteers were included. Demographics and measured parameters are shown in Table 1.

**Table 1:** Demographics/measured parameters of studied subjects.

Participants (n)	409
Age (Mean ± SD, years)	67.0 ± 5.5
Body height (Mean ± SD, cm)	151.6 ± 5.9
Body weight (Mean ± SD, kg)	56.0 ± 8.7
Radiographic parameters	
Thoracic kyphosis (Mean ± SD, degree)	29.8 ± 13.4
Lumbar lordosis (Mean ± SD, degree)	38.8 ± 16.0
Pelvic tilt (Mean ± SD, degree)	24.6 ± 11.0
Pelvic incidence (Mean ± SD, degree)	53.0 ± 10.6
Sagittal vertical axis (Mean ± SD, cm)	2.3 ± 3.6
Number of Schwab-SM (Mean ± SD)	2.1 ± 1.8
Non-radiographic: Muscle strength	
Trunk extension (Mean ± SD, BW ratio)	0.90 ± 0.33
Trunk flexion (Mean ± SD, BW ratio)	0.48 ± 0.15
Quadriceps femoris (Mean ± SD, BW ratio)	0.45 ± 0.19
Gluteus maximus (Mean ± SD, BW ratio)	0.38 ± 0.19
Iliopsoas (Mean ± SD, BW ratio)	0.17 ± 0.17
Non-radiographic Range of motion	
Hip extension (Mean ± SD, degree)	23.4 ± 6.6
Hip internal rotation (Mean ± SD, degree)	37.4 ± 8.8
Hip external rotation (Mean ± SD, degree)	37.6 ± 8.7
Knee flexion (Mean ± SD, degree)	149.8 ± 9.6
Knee extension (Mean ± SD, degree)	0.1 ± 4.7
Ankle dorsiflexion (Mean ± SD, degree)	26.0 ± 5.6
Back extension test (Mean ± SD, cm)	11.6 ± 5.7
Prone-press extension (Mean ± SD, cm)	27.0 ± 5.9
Ambulatory kyphosis (dTIA, Mean ± SD, degree)	4.5 ± 3.5
Back pain VAS (Mean ± SD, mm)	22.4 ± 24.0
Knee pain VAS (Mean ± SD, mm)	19.5 ± 18.7
SF36 PCS (Mean ± SD)	33.5 ± 6.5

Schwab-SM: Scoliosis Research Society and Schwab classification sagittal modifier; dTIA: difference of trunk inclination angle between rest and walk; VAS: visual analogue scale; SF36 PCS: short-form 36 physical component scale.

**Figure 4:**
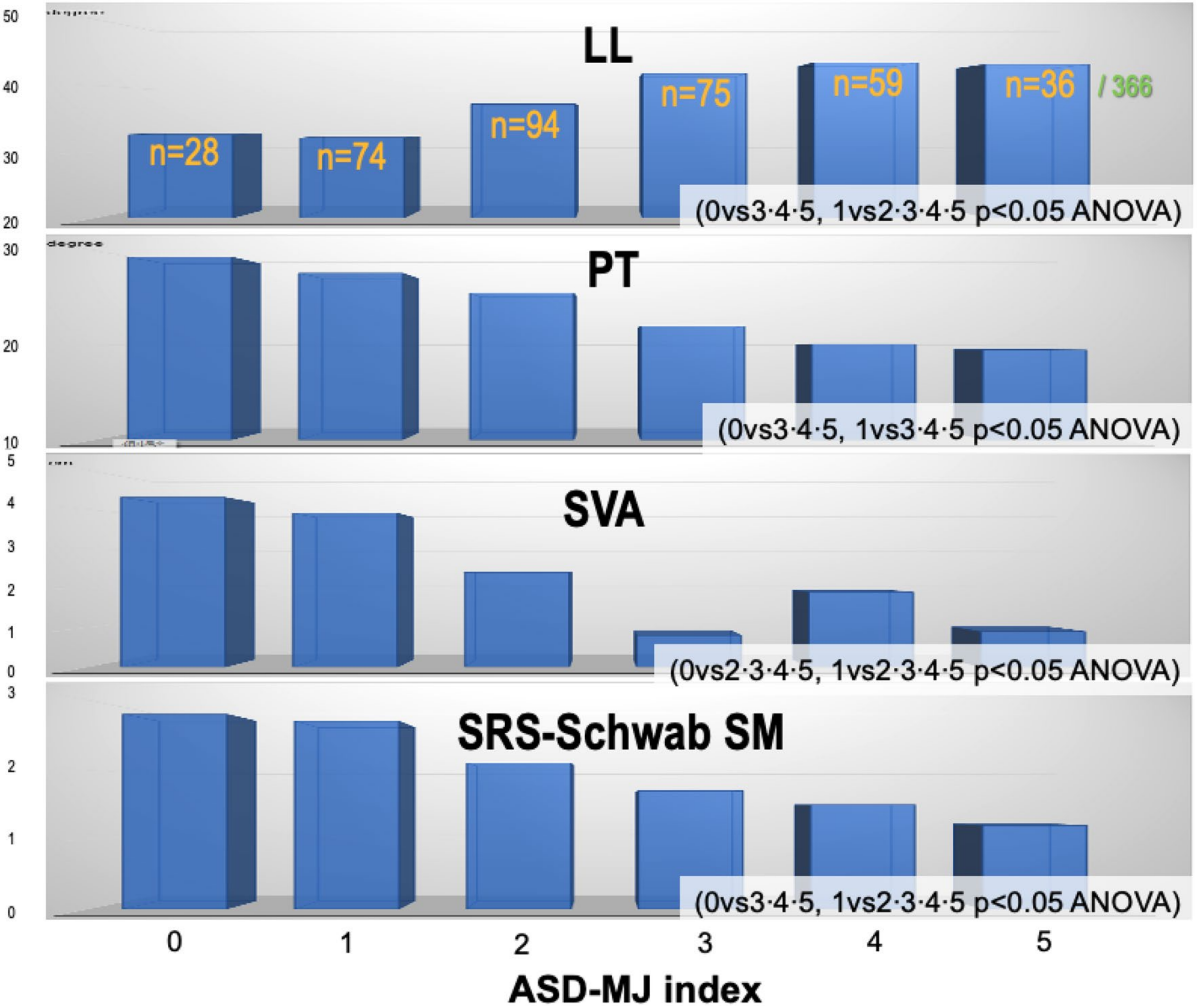
ASD-MJ & Radiographic. LL: lumbar lordosis. PT: pelvic tilt. SVA: sagittal vertical axis. SRS-Schwab SM: sagittal modifiers. ASD-MJ: adult spinal deformity muscle and joint factor. ANOVA analysis of variance.

**Figure 5:**
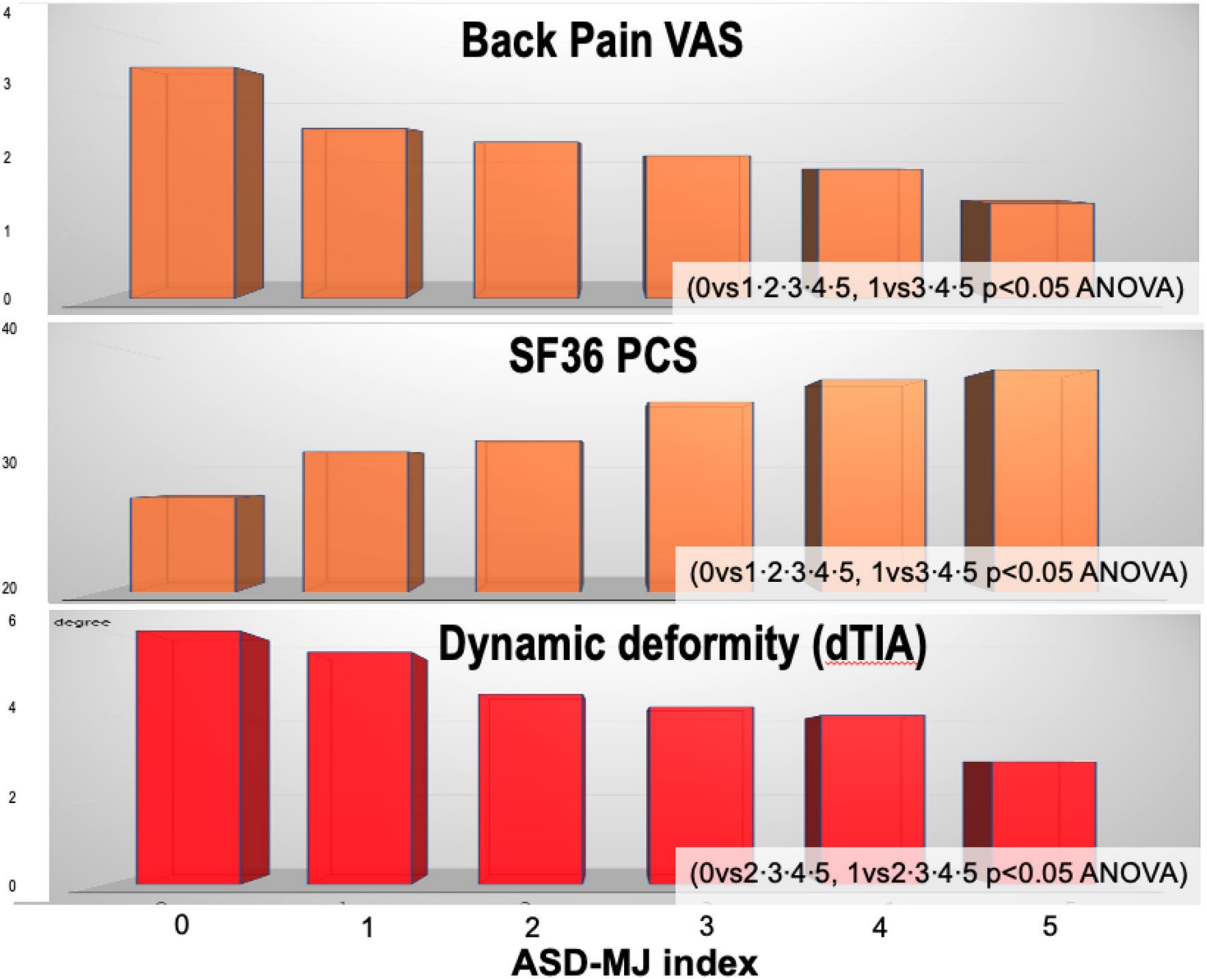
ASD-MJ & Non-radiographic parameters. VAS: visual analogue scale. SF36 PCS: physical component scale. dTIA: difference in trunk inclination angle. ASD-MJ: adult spinal deformity muscle and joint factor. ANOVA analysis of variance.

According to our previous study^6^, subjects with dTIA ≥ 7.6° were defined as pathological ambulatory kyphosis (n = 61), and dTIA ≤ 3.5° was defined as normal gait posture (n = 171). Using mean and standard deviation (SD) values of PCS (mean 33.5 ± 6.5), we defined clinical ASD (cASD) as PCS ≤ 27 (33.5–6.5) and pathological ambulatory kyphosis with dTIA ≥ 7.6° (n = 10). Robust counterpart were defined as PCS ≥ 40 (33.5 + 6.5) and dTIA ≤ 3.5° (n = 10), and non-cASD were defined as 27 < PCS < 40 and 3.5° < dTIA < 7.6° (n = 338). Table 2 shows statistical comparison of non-radiographic parameters among subjects with cASD, non-cASD, and robust counterpart, which revealed significant decrease in TEX, TFL, QF, KEX and BET in cASD subjects. Adopting normative data from robust subjects; (1) TEX ≥ 1.1BW, (2) TFL ≥ 0.5BW, (3) QF ≥ 0.5BW, (4) KEX ≥ 0°, (5) BET ≥ 14 cm, were defined as ASD-MJ index; 5-point muscle/joint goals for ASD (Table 3). ASD-MJ showed significant almost-linear correlation with radiographic and non-radiographic parameters (Figs. 4 and 5). ASD-MJ showed significant association with LL, PT, SVA and the number of Schwab sagittal modifiers, and the more the ASD-MJ index, the better the radiographic parameters. The same relation was found in clinical parameters.

**Table 2:** Statistical comparison among cASD, non-cASD, and robust group.

	cASD (n = 10)	Non-cASD (n = 338)	Robust (n = 19)	p-value (ANOVA)
TEX(BW ratio)	0.59	0.91	1.12	0.0002
TFL (BW ratio)	0.37	0.48	0.51	0.0276
QF (BW ratio)	0.36	0.50	0.55	0.0009
KEX (degree)	− 9.0	− 0.1	5.3	0.0001
BET (cm)	9.1	11.5	14.4	0.0347

cASD: clinical adult spinal deformity (dTIA ≥ 7.6° and PCS ≤ 27); Robust: (dTIA ≤ 3.5° and PCS ≥ 40); TEX: trunk extension; TFL trunk flexion; QF: quadriceps femoris; KEX: knee extension; BET: back extension test; BW: body weight; ANOVA: analysis of variance.

**Table 3:** ASD-MJ index (worst = 0/best = 5).

1) TEX	≥ 110%BW
2) TFL	≥ 50%BW
3) QF	≥ 50%BW
4) KEX	≥ 0°
5) BET	≥ 14 cm

ASD-MJ: adult spinal deformity muscle and joint factors; TEX: trunk extension; TFL: trunk flexion; QF: quadriceps femoris; KEX: knee extension; BET: back extension test; BW: body weight.

## Discussions

Recent multicenter studies indicated unsuccessful conservative treatment for ASD, and the mainstay of treating ASD has been surgery.

Acaroglu reported 1-year outcomes of 164 instrumented fusion cases and 371 conservative treatment cases of ASD, and indicated favorable outcomes were found in 42% of surgical cases and 6.7% of conservative cases. They showed that in most cases, the conservative treatment were mere observation and prescriptions, while 12 patients underwent physical therapy and 6 patients received invasive interventions such as injection therapy^3^. Glassman reported 5-year follow-up of 122 instrumented fusion cases and 73 conservative cases of ASD, and despite revision surgery in 24% and utilization of substantial resources in fusion cases, as-treated cost-effectiveness analysis favored surgery using the cumulative incremental cost-effectiveness ratio^1^.

Hoevenaars reported outcome of combined physical and psychological treatment for ASD patients (n = 80) and non-ASD patients (n = 240) with chronic low back pain. They showed that the ASD patients improved clinically as much as the non-ASD patients^10^. Hongo studied 102 Japanese women and reported that back extensor strength was significantly associated with LL and recommended back strengthening exercise for patients with kyphotic deformity^11^. Other studies also indicated the importance of back muscles, however, appropriate muscle load or targeted strength for each patient needed to be clarified. Proposed non-radiographic items, ASD-MJ, showed significant relation with radiographic and clinical outcomes, and subjects with back extensor strength of above 110% of BW, abdominal muscle strength of above 50% of BW, quadriceps strength of above 50% of BW, active back extension from prone position reaching 14 cm, and without knee flexion contractures were associated with less radiographic deformity and less back pain scores.

Limitation of our study included small number of subjects for heterogenic spinal deformity, yet this study included the largest-ever number with both radiographic and physical parameters including muscle strength and ROM. ASD-MJ was introduced using characteristic dynamic deformity and health-related quality of life (HRQoL) score, and showed significant relation with radiographic deformity and the number of Schwab-SM, however, this relation should be investigated further in interventional designs to confirm physical treatment effects for ASD. As an introductory for non-radiographic evaluation, this paper used the most popular Schwab-SM as radiographic parameters. Impact of coronal, axial, and other forms of ASD should be investigated in the future. Current ASD-MJ, using normative data from robust group, might be too strict to achieve, and data from non-cASD subjects, TEX ≥ 0.9BW, QF ≥ 0.5BW, KEX ≥ 0°, and BET ≥ 12 cm, provided comparable results and could be alternative goals for elderly patients with spinal deformity.

In conclusion, modifiable clinical goals should be clarified upon prescribing conservative or physical therapies, and our results suggested improving trunk and thigh muscle strengths, and lumbar and knee ROMs could lead to improved HRQoL and radiographic ASD scores. Demonstrated ASD-MJ index could be a benchmark for successful comprehensive management of ever-increasing patients of ASD.

## Date availability

Since the data for the current study included detailed demographics, such as age at each visit, body height and body weight, we decided not to share our data, and we would provide necessary data upon request. The data generated and/or analyzed during the current study are available from the corresponding author. (Kozaburo Mizutani, MD., E-MAIL: kyokui090096@gmail.com).
